# The role of MMP-1 in breast cancer growth and metastasis to the brain in a xenograft model

**DOI:** 10.1186/1471-2407-12-583

**Published:** 2012-12-07

**Authors:** Hui Liu, Yukinari Kato, Stephanie A Erzinger, Galina M Kiriakova, Yongzhen Qian, Diane Palmieri, Patricia S Steeg, Janet E Price

**Affiliations:** 1 Department of Cancer Biology, The University of Texas, M. D. Anderson Cancer Center, Houston, TX, 77030, USA; 2 Women’s Cancers Section, Laboratory of Molecular Pharmacology, National Cancer Institute, Bethesda, MD, 20892, USA

**Keywords:** Breast cancer, Brain metastasis, MMP-1, TGFα, EGFR

## Abstract

**Background:**

Brain metastasis is an increasingly common complication for breast cancer patients; approximately 15– 30% of breast cancer patients develop brain metastasis. However, relatively little is known about how these metastases form, and what phenotypes are characteristic of cells with brain metastasizing potential. In this study, we show that the targeted knockdown of MMP-1 in breast cancer cells with enhanced brain metastatic ability not only reduced primary tumor growth, but also significantly inhibited brain metastasis.

**Methods:**

Two variants of the MDA-MB-231 human breast cancer cell line selected for enhanced ability to form brain metastases in nude mice (231-BR and 231-BR3 cells) were found to express high levels of matrix metalloproteinase-1 (MMP-1). Short hairpin RNA-mediated stable knockdown of MMP-1 in 231-BR and 231-BR3 cells were established to analyze tumorigenic ability and metastatic ability.

**Results:**

Short hairpin RNA-mediated stable knockdown of MMP-1 inhibited the invasive ability of MDA-MB 231 variant cells in vitro, and inhibited breast cancer growth when the cells were injected into the mammary fat pad of nude mice. Reduction of MMP-1 expression significantly attenuated brain metastasis and lung metastasis formation following injection of cells into the left ventricle of the heart and tail vein, respectively. There were significantly fewer proliferating cells in brain metastases of cells with reduced MMP-1 expression. Furthermore, reduced MMP-1 expression was associated with decreased TGFα release and phospho-EGFR expression in 231-BR and BR3 cells.

**Conclusions:**

Our results show that elevated expression of MMP-1 can promote the local growth and the formation of brain metastases by breast cancer cells.

## Background

Breast cancer is the most common malignancy in women in the USA, and the second cause of cancer deaths after lung cancer; metastasis is the major cause of morbidity and mortality in breast cancer patients. Brain metastasis is an increasingly common complication in breast cancer patients, possibly a consequence of improvements in systemic therapies. Approximately 15-30% of breast cancer patients develop brain metastasis, with highest frequencies in patients with “triple-negative” or basal tumors, and also HER-2 positive tumors [1,2]. Investigations using patient samples [3] and xenograft model systems of brain metastasis [4,5] are leading to improved understanding of the pathobiology of brain metastasis.

Experimental models created to study the process of brain metastasis were used to isolated variants of the MDA-MB-231 human breast cancer cell line with enhanced brain metastatic ability. These selected variants have been used to identify and investigate the function of various genes contributing to the development of brain metastasis [6,7]. One gene, Matrix metalloproteinase-1 (MMP-1), was found to be highly expressed in two independently isolated variants of this cell line. Matrix metalloproteinases (MMPs) are a family of zinc-dependent endopeptidases which hydrolyze components of the extracellular matrix (ECM). Physiologically, these enzymes play a pivotal role in normal tissue re-modeling events such as in embryonic development, angiogenesis, ovulation, mammary gland involution and wound healing [8]. Moreover, high expression MMPs has been linked to several pathologies, including cancer invasiveness. Evidence from many clinical studies prompts further investigation of the pathophysiologic role of MMP-1 in metastatic progression. Increased MMP-1 expression has been associated with the incidence or invasiveness of various types of cancer, including colorectal, esophageal, pancreatic, gastric, breast, and malignant melanoma [9-13]. Furthermore, elevated MMP-1 expression in atypical ductal hyperplastic tissues may serve as a marker for predicting which patients will develop invasive breast cancer [14]. In addition to functions in tissue remodeling, tumor progression, and metastasis through its proteolytic activities for extracellular matrix (ECM) degradation, invasion, and cytokine mobilization [15], MMP-1 may also promote tumor invasion through proteolytic activation of the G protein coupled receptor PAR1 [16]. MMP-1 has also been shown to liberate signaling molecule precursors, such as pro-TGFα, other EGF-like ligands and TGFβ from cell surfaces or matrix [17-20]. This function may act to drive autocrine or paracrine signaling within the tissue environment, such that MMP-1 can contribute to angiogenesis or osteoclast activation [21,22]. In contrast to these well characterized functions of MMP-1 in tumor progression, its role in brain metastasis has received less attention.

In this study, we show that the targeted knockdown of MMP-1 in 231-BR and 231-BR3 cells not only reduced primary tumor growth, but also significantly inhibited the invasiveness of these two brain-seeking metastatic breast cancer cells and attenuated formation of experimental brain metastases. Inhibited MMP-1 expression also decreased TGFα release and phospho-EGFR expression in 231-BR and 231-BR3 cells. These results suggest that targeting MMP-1 and TGFα/EGFR signaling may be effective therapeutic strategies for breast cancer brain metastasis.

## Methods

### Cell lines

The 231-BR and 231-BR3 cells were derived from experimental brain metastases in nude mice injected with the MDA-MB-231 human breast cancer cell line, as reported previously [6,7]. The cells were maintained as monolayer cultures in MEM supplemented with 5% FBS, L-glutamine, MEM-vitamins, non-essential amino acid, sodium pyruvate, and puromycin for transduced cells (see below). Cell lines were validated by STR DNA fingerprinting using the AmpFℓSTR Identifiler kit according to manufacturer instructions (Applied Biosystems cat 4322288). The STR profiles were compared to known ATCC fingerprints (ATCC.org), to the Cell Line Integrated Molecular Authentication database (CLIMA) version 0.1.200808 (http://bioinformatics.istge.it/clima/) (Nucleic Acids Research 37:D925-D932 PMCID: PMC2686526) and to the MD Anderson fingerprint database. The STR profiles matched known DNA fingerprints for MDA-MB-231 human breast cancer cells.

### Generation of knockdown cells

Stable shRNA-mediated knockdown was achieved using SMARTvector shRNA lentiviral particles (with puromycin as selection marker) (Thermo Scientific Co) targeting the following sequences: sh1: GAGTACAACTTACATCGTG; sh2: GAACTGTGAAGCATATCGA; sh3: ACAGAATGTGCTACACGGA. A non-targeting control SMART vector was transduced as a shRNA control. shRNA lentiviruses were used to infect 231-BR and 231-BR3 cells in the presence of 5 μg/mL polybrene. The infected cells were selected with puromycin-supplemented (1 μg/ml) MEM. Surviving cells were expanded and analyzed for MMP-1 mRNA expression and protein expression. In this study, sh3 shRNA showed minor effect on knocking down MMP-1 (data not shown). shRNA lentiviruses targeting sh1 and sh2 sequences were used to infected 231-BR and 231-BR3 cells. Two pools of selected 231-BR cells infected with shRNA lentiviruses targeting sh1 sequence were named sh1a and sh1b. Two pools of selected 231-BR3 cells infected with shRNA lentiviruses targeting sh1 and sh2 sequences were named sh1 and sh2 respectively.

### RNA-isolation and real-time RT-PCR

Total RNAs from different cell lines and xenograft tumors were isolated with TriReagent (Sigma), treated with TURBO DNAse (Ambion), and reverse transcribed to cDNA with high capacity DNA archive reagents (Applied Biosystems) according to the manufacturer’s instruction. Real-time RT-PCR for MMP-1 was performed in duplicates of each sample using a total reactive volume of 25 μl, which contained 1.25 μl of 20× Gene Expression Assay Mix (Applied Biosystems), 12.5 μl of 2 × TaqMan Universal PCR Master Mix (Applied Biosystems) and 200 ng of cDNA template (diluted in RNase-free water to 11.25 μl). After 2 min at 50°C and 10 min at 95°C, 40 cycles of 15 s at 95°C and 1 min at 60°C were run. 18S in each sample was tested as intrinsic positive control. Each plate included at least three “No 7Template Controls (NTC)”. Reactions were run using the 7500 Real-Time PCR System (Applied Biosystems) and fluorescent data were converted into cycle threshold (ΔCT) measurements.

### ELISA

MMP-1 and TGFα protein expression levels were measured with an MMP-1 ELISA kit (Calbiochem Cat# QIA55) and TGFα Duoset ELISA development kit (R&D system Cat# DY239) according to the manufacturers’ instructions. To prepare samples for ELISA, cells were grown to 80% confluence with in medium with 0% or 5% FBS; supernatants from 24 h incubation were collected, and concentrated 10-fold, using Amicon ultra-4 centrifugal filters. MMP-1 and TGFα amounts were calculated as ng/ml and pg/ml protein, respectively, for different cell lines. For experiments using an MMP-1 inhibitor (EMD Chemicals, Gibbstown, NJ; cat# 444250) cells were incubated for 24 h with 2 μM of the inhibitor.

### Immunoblotting

Cells were harvested in RIPA lysis buffer, as described previously [23]. Proteins from total cell lysates or aliquots of concentrated conditioned medium, were resolved by the 7-12% Bis-Tris gradient gel, transferred to the pure nitrocellulose membrane, blocked in 5% non-fat milk or 5% BSA in TBS/Tween-20, and blotted with the antibodies for MMP-1 (1:1000, Millipore Cat# AB8105), phospho-EGFR (Tyr1068) (1:1000, Cell Signaling Cat# 2236), total EGFR (1:2000, Upstate Cat# 06–847), and β-actin (1:4,000, Sigma Cat# A2066).

### In vitro migration and invasion assay

For Transwell migration assays, 2.5×10^4^ cells were plated in the top chamber with the non-coated membrane (24-well insert; pore size, 8 μm; BD Biosciences Cat# 354578). For invasion assays, 2.5×10^4^ cells were plated in the top chamber with Matrigel-coated membrane (24-well insert; pore size, 8 μm; BD Biosciences Cat# 354480). In both assays, cells were plated in medium without serum or growth factors, and medium supplemented with serum (5% FBS) was used as a chemoattractant in the lower chamber. The cells were incubated for 24 h and cells that did not migrate or invade through the pores were removed with cotton swabs. Cells on the lower surface of the membrane were fixed and stained with the Fisher HealthCare PROTOCOL Hema 3 Manual Staining System (Fisher Scientific Co.) and counted.

### Tumorigenesis studies in mice

Six-week-old, specific pathogen-free athymic NCr-nu/nu mice were purchased from Charles Rivers or from the Animal Production Area of the National Cancer Institute-Frederick Cancer Research and Development Center (Frederick, MD). The care and use of laboratory animals was in accordance with the principles and standards set forth in the Principles for Use of Animals (NIH Guide for Grants and Contracts), the Guide for the Care and Use of Laboratory Animals, the provisions of the Animal Welfare Acts, and all procedures were approved by the Institutional Animal Care and Use Committees.

Parental MDA-MB-231 cells, BR3, BR and pooled stable knockdown cell lines containing the non-targeting vector or the MMP-1 shRNA (5×10^6^ cells/100 μl PBS) were injected into the mammary fat pad of nude mice, as described previously [23]. Tumors were measured weekly and tumor volume was calculated using the formula: volume = 0.5a^2^b (mm^3^), (a = smaller diameter, b = larger diameter).

MMP-1 shRNA and control shRNA expressing BR cells (shNTC, sh1a and sh1b) were injected into the left heart ventricle of nude mice (1.75×10^5^ cells in 0.1 ml PBS), as described previously [24]. Mice were euthanized under CO_2_ asphyxiation after 4 weeks, and brains were excised and immediately frozen in ornithine carbamyl transferase compound. Brain sections (10 μm thick) were serially cut every 300 μm and processed for hematoxylin and eosin (H&E) staining, and viewed using a microscope with 5 × objective and ocular grid with 0.8 mm^2^ squares. Numbers of metastases were counted in 10 sections from each brain, with micrometastases classified as lesions of < 300 μm, and large metastases as those that measured > 300 μm in any dimension. Two separate experiments were performed, and the data were combined for statistical analysis.

MMP-1 shRNA and control shRNA expressing BR3 cells (shCtr and sh1) were injected into tail veins of nude mice at the density of 2.5×10^5^ in 0.1 ml PBS for each cell line. Mice were sacrificed after 9 weeks and lungs were excised and fixed in formalin and processed for H&E staining. Lung metastases were counted on one H&E stained lung section from each mouse and classified into small (diameter < 0.5 mm), medium (diameter 0.5-1.5 mm) and large (diameter > 1.5 mm) metastases.

### Immunohistochemistry

Mammary fat pad tumors and lungs were collected from each mouse at necropsy, and fixed in 10% buffered formalin. Tissues were paraffin embedded, sectioned, and stained with H&E, MMP-1 (Epitomics Cat# 1973–1) and Ki-67 (Epitomics Cat# 4203–1). Brain frozen sections were stained with H&E and Ki-67 (Thermo/lab Vision Cat# RB-90-43) staining.

### Statistical analysis

Data are presented as mean ± SEM. Student’s *t* test (two tailed) was used to compare two groups (*P* < 0.05 was considered significant) unless otherwise indicated (Fisher’s exact test and ANOVA with Dunnett’s multiple comparison test). Microsoft Excel and Graphpad Prism software were used for statistical analyses.

## Results

### Stable expression of MMP-1 shRNAs knocks down MMP-1 expression in breast cancer cells

**Figure 1:**
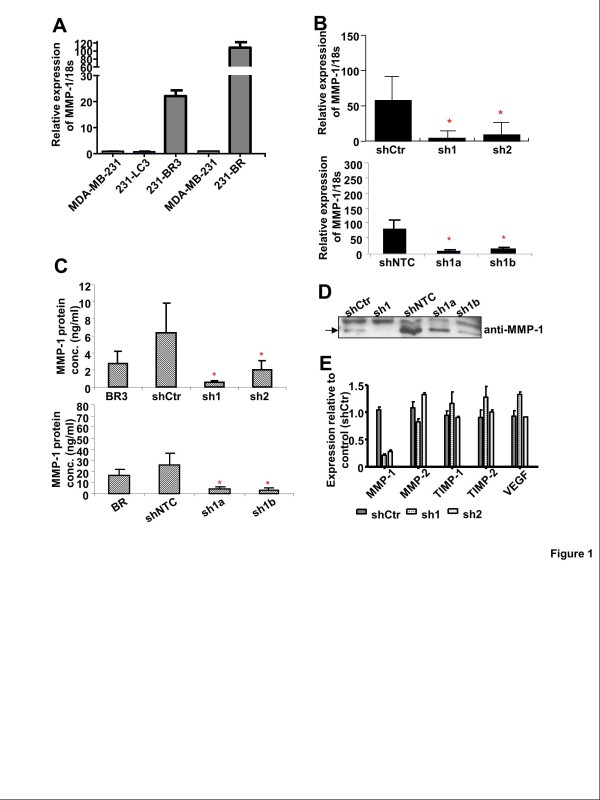
**MMP-1 shRNAs specifically inhibit MMP-1 expression in BR3 and BR cells. A**, real-time PCR quantification of MMP-1 mRNA levels. Compared with MDA-MB-231 parental cells and variants LC3 (selected from experimental lung metastases), the brain metastasis-derived BR3 and BR cells showed high levels of MMP-1 expression. This data shown are representative of 3 independent experiments. **B**, MMP-1 shRNAs knocked down MMP-1 mRNA level in BR3 and BR cell lines, compared with shCtr and shNTC cells expressing control shRNA. This experiment was performed in triplicate. **C**, ELISA assays and **D**, western blotting showed that MMP-1 protein levels in conditioned media were significantly reduced in BR3 and BR expressing MMP-1 targeting shRNA. These experiments were performed in triplicate. **E**, MMP-1 shRNA did not affect the expression of MMP-2, TIMP-1, TIMP-2 and VEGF in sh1 cells. This data shown are representative of 3 independent experiments.

Two variants of the MDA-MB-231 breast cancer cell line, 231-BR and 231-BR3, were established independently by two research groups, and have been shown to have enhanced brain-metastasizing potential [6,7]. Microarray analyses were performed by the Steeg laboratory to identify common differentially expressed genes; altered expression of 26 genes was seen in both brain metastasis-derived variants compared with the parental cell line. Of these, MMP-1 was the most highly expressed gene. The expression of MMP-1 gene in 231-BR cells increased 89-fold and in 231-BR3 cells increased 36-fold compared with parental MDA-MB-231 cells (data not shown). The increased expression was confirmed using real time PCR measurements (Figure 1A). Included in the comparison was a variant selected from experimental lung metastases (231-LC3 [6]), which did not express increased MMP-1; this suggested that the increase in expression is not a consequence of selection of cells from xenografted tumors in general, but may be linked to the formation of experimental brain metastases.

Silencing MMP-1 expression in 231-BR and 231-BR3 cells was undertaken to define the role of MMP-1 in brain metastasis. Three different sequence-targeting short hairpin RNA (shRNA) lentiviral particles were transfected into 231-BR and 231-BR3 breast cancer cells. Cells were selected with puromycin-supplemented (1 μg/ml) MEM. Surviving cells were expanded and analyzed for MMP-1 mRNA expression and protein expression. Stably MMP-1 knockdown cell lines sh1, sh2 (231-BR3) and sh1a, sh1b (231-BR) showed decreased MMP-1 mRNA expression (Figure 1B). ELISA and immunoblots of culture supernatants showed that secreted MMP-1 protein was reduced in samples collected from the shRNA-expressing cell lines compared with control cell lines shCtr (231-BR3) and shNTC (231-BR), respectively (Figure 1C, D). Cell lines transfected with lentivirus with the sh3 sequence showed no reduction in MMP-1 expression, and were not used for further experiments.

The specificity of MMP-1 shRNA was determined by measuring the relative expression of MMP-2 and MMP-7; no expression of the latter was detected. Transduction with shRNA to MMP-1 did not substantially alter expression of MMP-2, TIMP-1, TIMP-2 or VEGF (Figure 1E shows data for 231-BR3 transfectants; the same experiments with 231-BR transfectants yielded similar results).

### MMP-1 suppression inhibits invasion ability of breast cancer cells in vitro

**Figure 2:**
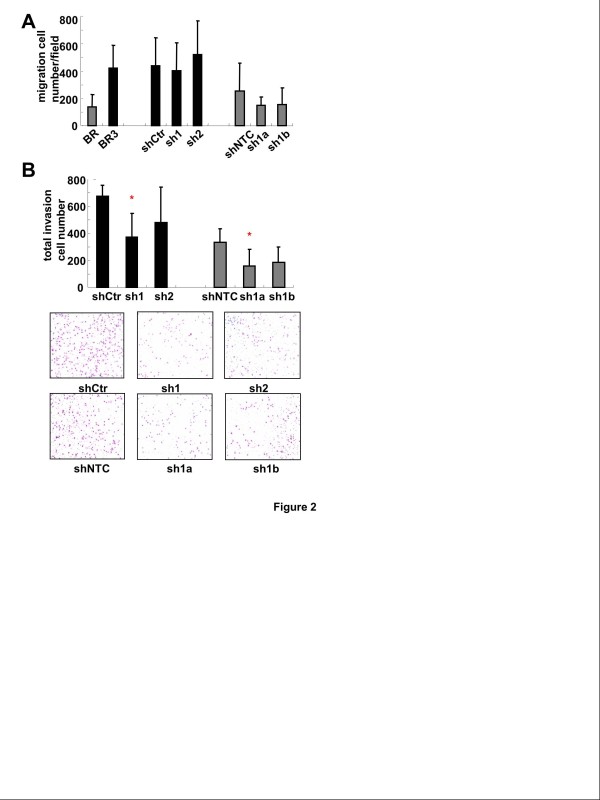
**MMP-1 suppression inhibits invasion ability but not migration ability of 231-BR3 and 231-BR cell lines *in vitro*. A**, 231-BR cells, 231-BR3 cells, control shRNA and MMP-1 shRNA expressing cells were tested for their ability to migrate to 5% FBS in the lower chamber of Transwell chambers. 2.5×10^4^ cells were seeded in the migration chamber in serum-free medium. Migrated cells were fixed and counted after 24 h. MMP-1 knockdown in BR and BR3 cells did not affect cell migration ability. The data shown were combined from 5 independent experiments. **B**, control shRNA and MMP-1 shRNA expressing BR and BR3 cells were tested for ability to invade across a Matrigel-coated membrane in response to 5% FBS in the lower chamber. 2.5×10^4^ cells were seeded in the invasion chamber in serum-free medium. Invaded cells were fixed and counted after 24 h. Asterisks indicate significant differences (*P* < 0.05) between control shRNA expressing cells and MMP-1 shRNA expressing cells. The data shown were combined from 5 independent experiments.

Recent studies showed that pericellular degradation of substrates by membrane-tethered MMPs is a key step for promoting cell invasion [20]. Having found elevated expression of MMP-1 in 231-BR cells and 231-BR3 cells, we sought to test whether MMP-1 shRNA could inhibit their invasiveness. First we tested if MMP-1 knockdown affected the motility of 231-BR and 231-BR3 cells. The results showed that MMP-1 knockdown in BR and BR3 cells did not affect cell migration ability (Figure 2A). Then control shRNA and MMP-1 shRNA expressing BR and BR3 cells were tested for ability to invade across a Matrigel-coated membrane in response to 5% FBS in the lower chamber. The result indicated that there was a significant reduction (*P* < 0.05) in the invasive properties of MMP-1 shRNA expressing cells compared with control shRNA expressing cells (Figure 2B). Taken together, these observations suggested that MMP-1 function is required for in vitro invasiveness but not for motility of these metastatic cells.

**Figure 3:**
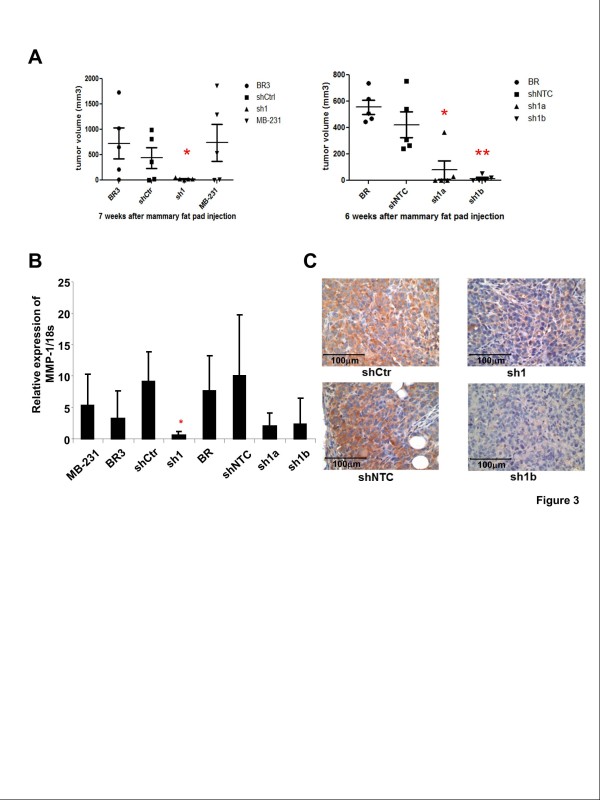
**Stable knockdown of MMP-1 expression inhibits local tumor growth. A**, each cell line was injected into mammary fat pads of nude mice (5×10^6^ cells per mouse). Each group includes five mice. Asterisks indicate that the tumors of MMP-1 knockdown cell line sh1 grew significantly slowly than the control cell line shCtr (*P* < 0.05, *t*-test) and the tumors of MMP-1 knockdown cell lines sh1a and sh1b grew significantly slowly than those of the control cell line shNTC (*P* < 0.01, ANOVA Dunnett’s Multiple comparison test). **B**, real-time PCR quantification of MMP-1 mRNA levels of tumors showed a significant reduction in tumors of sh1 cells compared with shCtr tumors. **C**, representative MMP-1 immunohistological staining of tumor sections showing reduced staining in sh1 and sh1b tumors.

**Figure 4:**
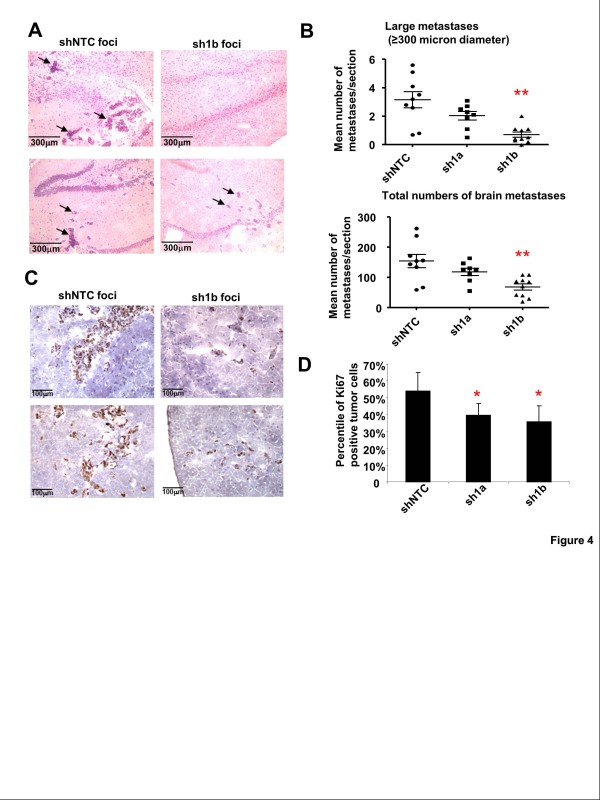
**MMP-1 knockdown in 231-BR cells attenuates brain metastasis.** MMP-1 shRNA expressing 231-BR cells, sh1a and sh1b, and control shNTC cells were injected into the left heart of nude mice, 1.75×10^5^ cells per mouse. Each group includes ten mice. Mice were sacrificed after 4 weeks and the number of experimental metastases scored in serial brain sections. **A**, Representative H&E staining of brain sections. Arrows indicate metastatic foci. **B**, The sh1a and sh1b cells formed fewer large metastases and total metastases compared with the control shNTC cells. The data were combined from two independent experiments, shown as the mean and SEM of metastases scored in serial sections. Asterisks indicate that sh1b cells formed significantly fewer large and total metastases compared with shNTC cells (*P* = 0.002, *P* = 0.0067 respectively, ANOVA Dunnett’s Multiple comparison test). **C**, Ki-67 stained sections of brain metastases formed by injection of 231-BR control cells and MMP-1 knockdown cells and **D**, comparisons of Ki-67 positive cells in brain metastases. Significantly fewer Ki-67 positive cells were found in brain metastases of sh1a (*P* = 0.022) and sh1b (*P* = 0.011, Student’s *t*-test) compared with metastases of shNTC cells.

**Figure 5:**
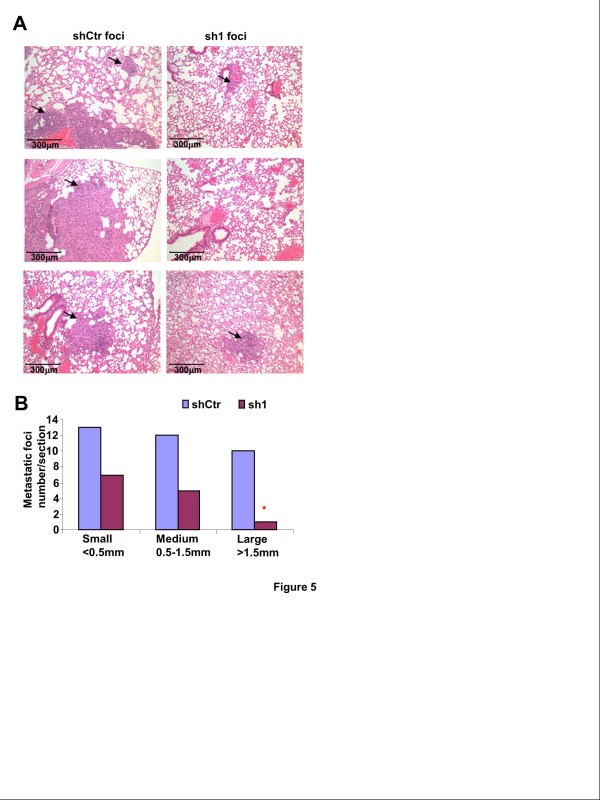
**MMP-1 knockdown in BR3 cells attenuates lung metastasis.** MMP-1 shRNA expressing 231-BR3 cells sh1 and control shCtr cells were injected into the tail veins of nude mice at the density of 2.5×10^5^/100 μl PBS. Each group includes ten mice. Mice were sacrificed and lung sections were analyzed after 9 weeks. **A**, representative H&E staining of lung sections. Arrows indicate metastatic foci. **B**, for each group, total lung metastatic foci were counted. Fisher’s exact test showed a significant difference between the numbers of lung foci in the sh1 group compared to shCtr group (*P* < 0.05).

**Figure 6:**
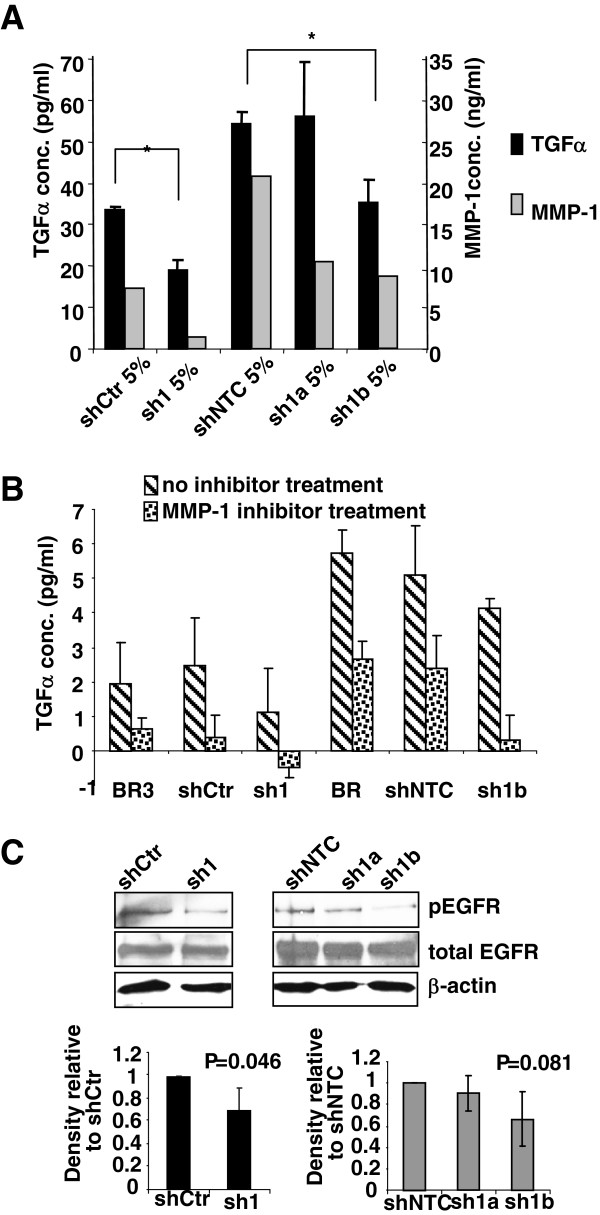
**Stable knockdown of MMP-1 is associated with reduced TGFα in culture supernatants. A**, MMP-1 and TGFα protein levels in conditioned media were measured by ELISA. Cells were cultured in MEM with 5% FBS to 80% confluence. Supernatants were separated and concentrated, and MMP-1 and TGFα protein concentrations were measured by ELISA. Asterisks indicate significant differences (*P* < 0.05) in TGFα in culture supernatants from control shRNA expressing cells compared with MMP-1 shRNA expressing cells. This data shown are representative of 3 independent experiments. **B**, TGFα concentrations in conditioned media were measured by ELISA after 24 h incubation with MMP-1 inhibitor at 2 μM, in serum free medium. This data shown are representative of 3 independent experiments. **C**, Immunoblotting for phospho-EGFR, total EGFR and β-actin protein levels in each cell line cultured in culture medium with 5% FBS. This experiment was performed in triplicate.

### Stable knockdown of MMP-1 expression inhibits local tumor growth

MTT assays were used to analyze if MMP-1 reduction affects breast cancer cell proliferation in vitro. The result showed no difference in proliferation between control and MMP-1 knockdown cells (Additional file 1). To test whether MMP-1 knockdown affected tumor growth *in vivo*, we injected MMP-1 shRNA expressing cells and control shRNA expressing cells into the mammary fat pads of nude mice. After 6 weeks, the tumors of MMP-1 knockdown cell lines sh1 and sh1b were significantly smaller than those of the control lines shCtr and shNTC (Figure 3A). Real-time PCR quantification using RNA isolated from tumor tissues confirmed the continued silencing of MMP-1 *in vivo* (Figure 3B). Immunohistochemical analysis of tumor sections also demonstrated reduced staining for MMP-1 in sh1 and sh1b tumors compared with tumors of the control cell lines (Figure 3C).

The incidence of tumor formation by the MMP-1 knockdown cells was moderately reduced compared with the control cell lines (231-BR-shNTC, 100% tumor take compared with 231-BR-sh1b, 67% tumor take; 231-BR3-shCtr, 80% tumor take, compared with sh1, 60%), although the differences were not statistically significant, using Fisher’s Exact test (Additional file 2). To determine whether MMP-1 knockdown affected cell proliferation in breast tumor, proliferation marker Ki-67 staining was performed on sections of breast tumor. The result showed no difference in proliferation between control and MMP-1 knockdown tumors (Additional file 3).

Taken together, although MMP-1 was not essential for tumor initiation in the mammary fat pad and its reduction has no effect on proliferation of breast cancer cells in vitro, silencing expression of MMP-1 reduced tumor growth *in vivo*.

### Inhibition of MMP-1 in 231-BR cells attenuates brain metastasis

A key question was whether MMP-1 could promote brain metastasis. To determine whether MMP-1 knockdown affected brain metastasis, 1.75×10^5^ MMP-1 knockdown cells (sh1a, sh1b) and control cells (shNTC) cells were injected into the left heart of nude mice, as described previously [24]. Mice were sacrificed 4 weeks later and brains removed for analysis of metastasis formation. The sh1a and sh1b cells formed fewer large metastases (reduced by 43% and 80.5%, respectively) and fewer total metastases (reduced by 31% and 43%, respectively) compared with the control shNTC cells (Figure 4B, A).

To determine whether MMP-1 knockdown affected cell proliferation in brain metastases, we performed immunohistochemistry with the Ki-67 proliferation marker. We found that the brain metastases of the shNTC cells had significantly more Ki-67 positive cells that the brain metastases of the MMP-1 knockdown cells (Figure 4C, D). Hence, MMP-1 knockdown reduced the proliferation of metastatic breast cancer cells in the brain.

### Inhibition of MMP-1 in 231-BR3 cells attenuates lung metastasis

We next injected MMP-1 shRNA expressing 231-BR3 cells (sh1) and control shCtr cells into the tail veins of nude mice. Mice were sacrificed and lung sections were analyzed after 9 weeks. Numerous metastatic nodules were observed in the lungs of mice inoculated with control shCtr cells, but fewer were found in the lungs of mice injected with sh1 cells (Figure 5A). To detect small metastatic foci, lung sections were stained with H&E, and the numbers of lung metastatic foci were counted and measured. Lung metastases were categorized into 3 groups based on the size; less than 500 μm of diameter was termed small, 0.5-1.5 mm, medium and greater than 1.5 mm of diameter, large. Fisher’s Exact test showed a significant difference in the incidence of large metastases in the lungs of mice injected with sh1 cells compared to samples from mice injected with shCtr cells (*P* < 0.05) (Figure 5B). This result further confirmed that MMP-1 expression promotes the development of metastases.

### Stable knockdown of MMP-1 expression is associated with reduced TGFα release and activation of EGFR

These data showed that reducing MMP-1 expression not only reduced local mammary tumor growth, but also attenuated the metastatic ability of breast cancer cells. In vitro, MMP-1 knockdown reduced the invasiveness of breast cancer cells. Many reports have shown that MMP-1 can promote tumor growth and metastasis through catalyzing extracellular matrix and by promoting angiogenesis [21]. In addition, MMP-1 can activate or release growth factors to promote metastasis [17,19,22].

To test if release of TGFα was linked to MMP-1 expression in the brain metastasis-derived variants of MDA-MB-231, we measured both MMP-1 and TGFα concentrations in culture supernatants of MMP-1 shRNA expressing cells and control cells by ELISA (Figure 6A). The results showed lower concentrations of TGFα in samples from the MMP-1 knockdown cell lines. The addition of an MMP-1 inhibitor in further experiments confirmed that MMP-1 activity can modulate levels of TGFα in culture supernatants of the breast cancer cell lines (Figure 6B). To confirm that the observed reduction in TGFα was not due to an off-target effect of the shRNA to MMP-1, real-time RT-PCR measurements of TGFαwere performed. The results showed no substantial change in expression of TGFα between the control and MMP-1 knockdown cell lines (Additional file 4).

As TGFα is a ligand for EGFR, we next measured phospho-EGFR expression in MMP-1 knockdown cells and control cell lines (Figure 6C). In the MMP-1 knockdown cells (sh1 and sh1b), the phospho-EGFR levels were lower than in control cells (shCtr and shNTC). The results demonstrated that active MMP-1 proteolysed latent TGFα to generate active TGFα, leading to an activated EGFR signal pathway, thus linking MMP-1 and the EGFR signaling pathway in metastatic breast cancer cells.

## Discussion

In this report we provide evidence that elevated expression of MMP-1 contributes to the brain colonizing potential of human breast cancer cells in xenograft models of cancer progression.

Members of the MMP family play important roles in normal and malignant processes. Their functions in invasion and metastasis have been associated primarily with degradation of ECM components [18,25,26]. In recent years, however, it has become increasingly clear that MMP substrates extend to many non-matrix extracellular and membrane-bound proteins, including protease precursors and inhibitors, cytokines, latent growth factors, growth factor-binding proteins, and adhesion molecules [17]. Understanding how MMP-1 and other members of the MMP family promote metastasis, in part by altering the signaling milieu in the tissue microenvironment colonized by disseminating cells may be crucial for developing more effective therapies for metastatic cancer.

Our study shows that MMP-1 is highly expressed by the brain metastasis-derived variants of the human MDA-MB-231 breast cancer cell line; this supports findings reported by others [4]. What drives the elevated expression remains to be established; the 231-BR and 231-BR3 variants have constitutive activation of STAT3, which has been linked to elevated expression of various genes, including VEGF, cyclin D and survivin [27]. Transcriptional regulators of MMP-1 in cancer cells include STAT3 [28] and members of the AP-1 family of transcription factors [29].

Reducing the expression of MMP-1 with shRNA attenuated tumor growth in the mammary fat pads and reduced invasion through matrix-coated filters of the 231-BR and 231-BR3 cells, similar to the findings of other investigators using non-selected MDA-MB-231 cells [30,31]. Metastatic lesions formed by cells expressing shRNA to MMP-1 in the lungs or brains, from i.v. or intra-cardiac injections, respectively, were smaller and fewer than those formed by the control cells. Without using a method to follow the fate of cells after injection into mice we cannot discern whether the reduction in metastasis number is due to impaired arrest and extravasation, or reduced proliferation in the metastatic site; the data would support a combination of these possibilities. MRI has been used to document the fate of MDA-MB-231-BR cells tagged with iron oxide particles in the brains of nude mice after intra-cardiac injection. The majority of the cells were rapidly eliminated, and only a small fraction of the initial inoculum formed actively growing metastases [32]. Fitzgerald *et al.*[33] reported high proliferation rates of brain metastases of this cell line, as we also found for metastases of control shRNA-expressing cells, while significantly fewer cells in brain metastases of the MMP-1 silenced cell lines were proliferating (Ki67-positive).

Our data show that MMP-1 can regulate the levels of TGF-α in culture supernatants of the MDA-MB-231-BR and -BR3 cells, which in turns affects activity of EGFR in the cancer cells. The activation of EGFR can regulate a wide variety of cellular functions [34,35]. One related to brain metastasis is the recent report of EGF promoting heparanase function and Topoisomerase I localization in brain metastasizing breast cancer cells [36]. Treatment with cetuximab, a humanized antibody to EGFR, reduced transmigration through a simulated blood–brain barrier and extended survival of mice injected with brain-colonizing breast cancer cells [4]. MDA-MB-231-BR cells express phosphorylated EGFR *in vivo*, and treatment with lapatinib, a small molecule tyrosine kinase inhibitor of EGFR and HER2, significantly reduced the numbers of large brain metastases formed by these cells [37]. The TGF-α released around MMP-1 expressing cells may also have paracrine functions in the brain microenvironment, including induction of angiogenesis and neurogenesis [38], and activation of astrocytes in response to injury [39,40]. Reactive microglial and astrocytic responses to brain metastases have been reported in studies using the MDA-MB-231-BR model [33] and other experimental brain metastasis models [41,42], resembling the peritumoral changes seen in clinical brain metastases [43]. These responses may promote the proliferation and survival of the metastatic cells [33,41,44,45]. Reactive astrocytes have neuroprotective functions, which may be exploited by cancer cells; co-culture of astrocytes with brain metastatic cells protected the latter from chemotherapy-induced apoptosis, an effect dependent upon gap-junction communications between the different cell types [5,42].

While not explored further in this study, MMP-1 activation of protease-activated receptor 1 (PAR1) may also contribute to the process of brain metastasis. Protease-activated receptors are members of the G protein coupled receptor family that are activated upon cleavage of an N-terminal tethered ligand. Thrombin and MMP-1 both activate PAR1, but MMP-1 is reported to cleave the tethered ligand at a unique site [46]. PAR1 expression on breast cancer cells has been associated with a high metastatic potential, and inhibiting the downstream signals from PAR1, using a small molecule inhibitor, suppressed Akt-mediated survival pathways, and attenuated tumor growth and experimental lung metastasis [47]. The brain metastatic variants of MDA-MB-231 maintain the high expression of PAR1 reported by others for the original cell line (Liu and Price, unpublished). PAR1 is also expressed by other cell types present in the brain microenvironment, including endothelial cells [48] and astrocytes; activation of PAR1 on the latter can trigger astrogliosis [49].

## Conclusions

Tumor metastasis is a complex and highly regulated process involving multiple tumor-host interactions, mediated by various host- and tumor-derived factors [50,51]. Our results, together with those from many other studies, suggest that blocking the actions of MMP-1 should theoretically prove beneficial in the treatment of invasive and metastatic cancers. However, clinical trials with broad-spectrum MMP inhibitors for various cancers have failed to improve patient outcome and often produced adverse events, including dose-limiting joint toxicity [52,53]. As more details of functions of MMP-1 in metastasis to the brain and other organs are defined, this information may be useful for decisions of clinical management. MMP-1 has been proposed as a biomarker for breast cancer [14,53]; understanding its role in activation of the TGFα/EGFR signal pathway may lead to the use or development of additional targeted agents to suppress this axis, and result in improved treatments for metastatic breast cancer.

## Abbreviations

MMP-1: Matrix metalloproteinase-1; ECM: Extracellular matrix; PAR1: Protease-activated receptor 1; TGF: Transforming growth factor; EGFR: Epidermal growth factor receptor; TIMP: Tissue inhibitor of metalloproteinases; VEGF: Vascular endothelial growth factor.

## Competing interests

The authors declare that they have no competing interests.

## Authors’ contributions

JEP and PS conceived the project. HL designed and performed experiments. JEP supervised research. KY established MMP-1 knockdown cell lines. SAE analysed data. GMK assisted with animal experiments. YQ and DP performed animal experiments and prepared frozen brain blocks. HL and JEP wrote manuscript. All authors read and approved the final manuscript.

## Pre-publication history

The pre-publication history for this paper can be accessed here:

http://www.biomedcentral.com/1471-2407/12/583/prepub

## Supplementary Material

Additional file 1 **Parental MDA-MB-231, BR3, BR and shRNA transfected variant cells were seeded in 96-well plates (100 μl/well) at a concentration of 1×106 cells/ml and cultured at 37ºC, 5% CO**_**2 **_**in MEM medium.** For time-dependent assays, cells were incubated for 24 h, 48 h and 72 h. Cell viability was analyzed using MTT (3 (4, 5-dimethylthiazol-2yl)-2, 5-diphenyltetrazolium bromide). Statistical comparison by Student’s *t*-test is expressed as p > 0.05 for both BR and BR3 groups. Click here for file

Additional file 2 **Parental MDA-MB-231, BR3, BR and shRNA transfected variant cells were injected into mammary fat pads of nude mice at an inoculum of 5X10**^**6 **^**cells/0.1 ml.** After 7 weeks, mice were sacrificed and tumor formation was compared. Statistical comparison by Fisher’s exact test is expressed as p > 0.05 for both BR and BR3 groups. Click here for file

Additional file 3 **Ki-67 staining was performed on sections of breast tumor induced by mammary fat pad injection of BR and BR3 control cells and MMP-1 knockdown cells.** A, representative staining images and B, quantification of Ki-67 staining. Click here for file

Additional file 4 Real-time PCR quantification of TGFα and EGFR mRNA levels of control cells (shCtr and shNTC) and MMP-1 knockdown cells (sh1 and sh1a, sh1b) in BR3 and BR cells. Click here for file
