# Supplementary figures and images for: The role of MMP-1 in breast cancer growth and metastasis to the brain in a xenograft model
Source: BMC Cancer. 2012 Dec 7;12:583. doi: 10.1186/1471-2407-12-583 (PMC3526403; doi:10.1186/1471-2407-12-583)

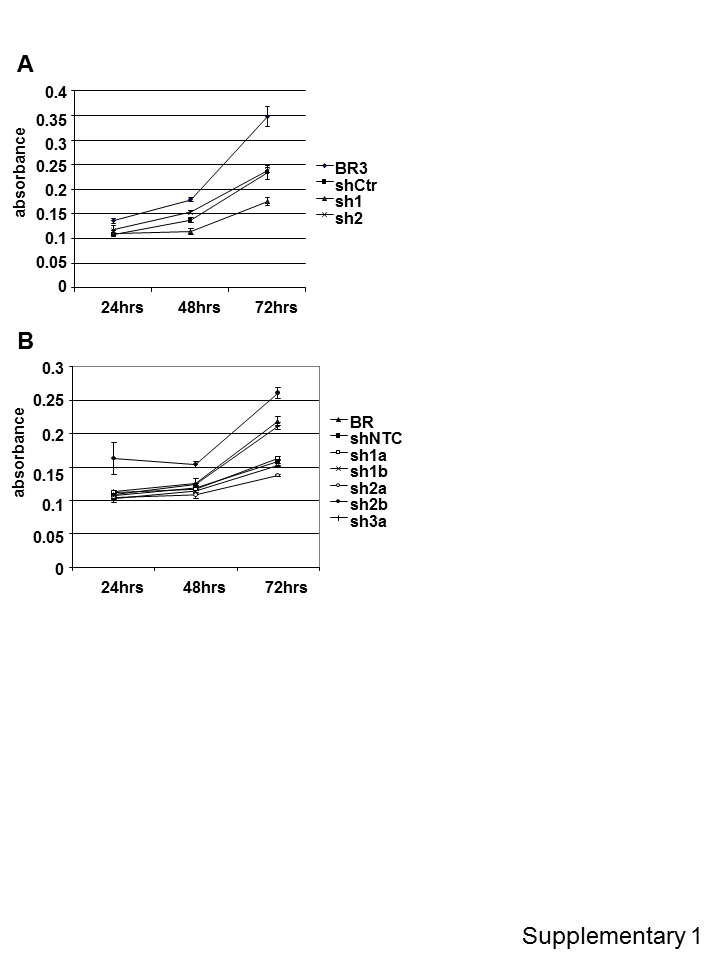

Supplement: Additional file 1 — Parental MDA-MB-231, BR3, BR and shRNA transfected variant cells were seeded in 96-well plates (100 μl/well) at a concentration of 1×10^6^ cells/ml and cultured at 37ºC, 5% CO2 in MEM medium. For time-dependent assays, cells were incubated for 24 h, 48 h and 72 h. Cell viability was analyzed using MTT (3 (4, 5-dimethylthiazol-2yl)-2, 5-diphenyltetrazolium bromide). Statistical comparison by Student’s t-test is expressed as p > 0.05 for both BR and BR3 groups. [file 1471-2407-12-583-S1.tiff]

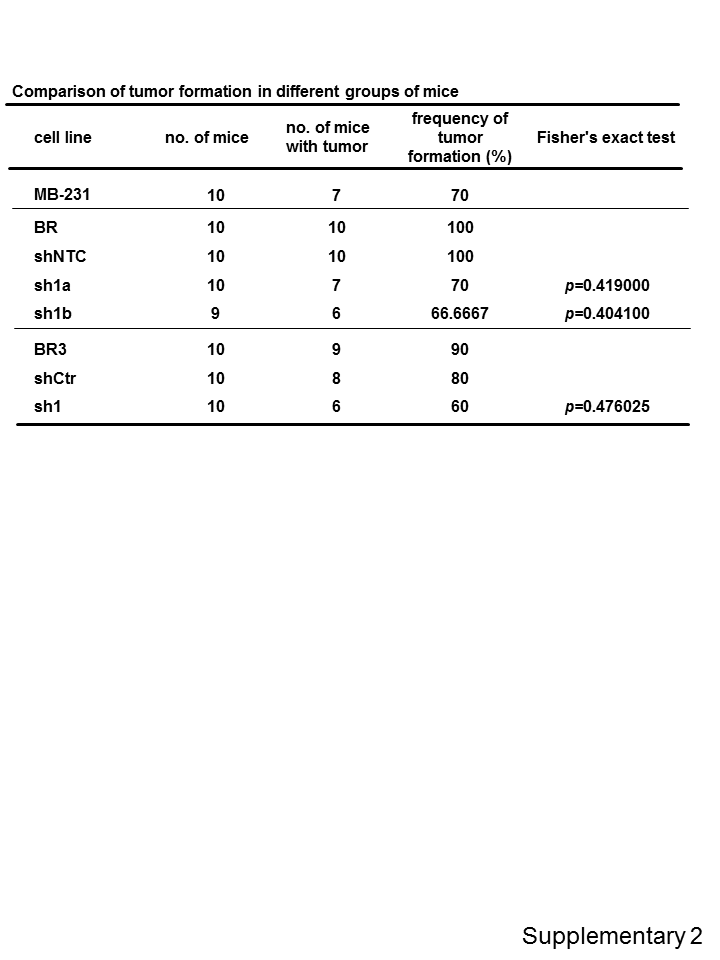

Supplement: Additional file 2 — Parental MDA-MB-231, BR3, BR and shRNA transfected variant cells were injected into mammary fat pads of nude mice at an inoculum of 5X106 cells/0.1 ml. After 7 weeks, mice were sacrificed and tumor formation was compared. Statistical comparison by Fisher’s exact test is expressed as p > 0.05 for both BR and BR3 groups. [file 1471-2407-12-583-S2.tiff]

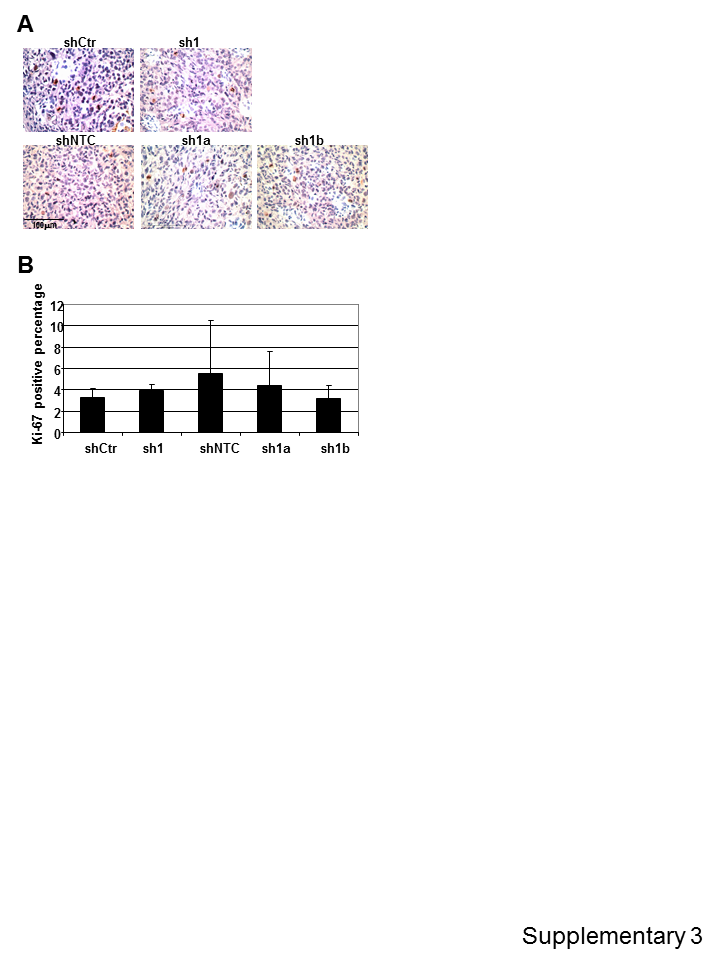

Supplement: Additional file 3 — Ki-67 staining was performed on sections of breast tumor induced by mammary fat pad injection of BR and BR3 control cells and MMP-1 knockdown cells. A, representative staining images and B, quantification of Ki-67 staining. [file 1471-2407-12-583-S3.tiff]

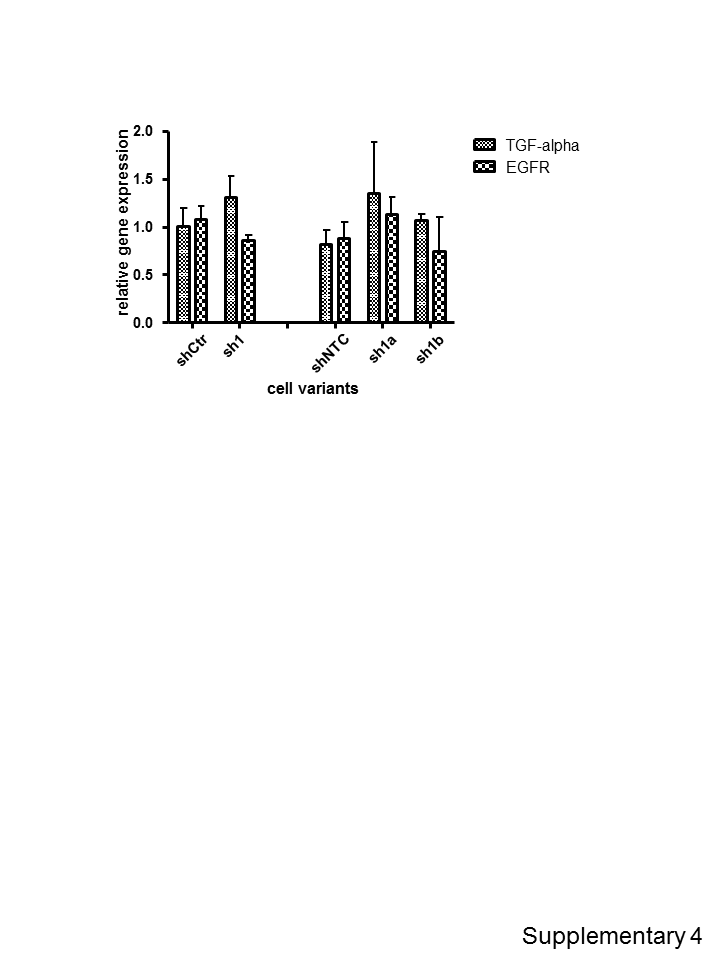

Supplement: Additional file 4 — Real-time PCR quantification of TGFα and EGFR mRNA levels of control cells (shCtr and shNTC) and MMP-1 knockdown cells (sh1 and sh1a, sh1b) in BR3 and BR cells. [file 1471-2407-12-583-S4.tiff]
